# Molecular weight determination of adeno‐associate virus serotype 8 virus‐like particle either carrying or lacking genome via native nES gas‐phase electrophoretic molecular mobility analysis and nESI QRTOF mass spectrometry

**DOI:** 10.1002/jms.4786

**Published:** 2021-10-04

**Authors:** Samuele Zoratto, Victor U. Weiss, Jerre van der Horst, Jan Commandeur, Carsten Buengener, Alexandra Foettinger‐Vacha, Robert Pletzenauer, Michael Graninger, Guenter Allmaier

**Affiliations:** ^1^ Institute of Chemical Technologies and Analytics TU Wien (Vienna University of Technology) Vienna Austria; ^2^ MS Vision Almere The Netherlands; ^3^ Pharmaceutical Sciences Baxalta Innovations (part of Takeda) Vienna Austria

**Keywords:** AAV, adeno‐associated virus, GEMMA, gene therapy platform, native MS

## Abstract

Virus‐like particles (VLPs) are proteinaceous shells derived from viruses lacking any viral genomic material. Adeno‐associated virus (AAV) is a non‐enveloped icosahedral virus used as VLP delivery system in gene therapy (GT). Its success as vehicle for GT is due to its selective tropism, high level of transduction, and low immunogenicity. In this study, two preparations of AAV serotype 8 (AAV8) VLPs either carrying or lacking completely genomic cargo (i.e., non‐viral ssDNA) have been investigated by means of a native nano‐electrospray gas‐phase electrophoretic mobility molecular analyzer (GEMMA) (native nES GEMMA) and native nano‐electrospray ionization quadrupole reflectron time‐of‐flight mass spectrometry (MS) (native nESI QRTOF MS). nES GEMMA is based on electrophoretic mobility principles: single‐charge nanoparticles (NPs), that is, AAV8 particle, are separated in a laminar sheath flow of dry, particle‐free air and a tunable orthogonal electric field. Thus, the electrophoretic mobility diameter (EMD) of a bio‐NP (i.e., diameter of globular nano‐objects) is obtained at atmospheric pressure, which can be converted into its M_W_ based on a correlation. First is the native nESI QRTOF. MS's goal is to keep the native biological conformation of an analyte during the passage into the vacuum. Subsequently, highly accurate M_W_ values are obtained from multiple‐charged species after deconvolution. However, once applied to the analysis of megadalton species, native MS is challenging and requires customized instrumental modifications not readily available on standard devices. Hence, the analysis of AAV8 VLPs via native MS in our hands did not produce a defined charge state assignment, that is, charge deconvolution for exact M_W_ determination was not possible. Nonetheless, the method we present is capable to estimate the M_W_ of VLPs by combining the results from native nES GEMMA and native ESI QRTOF MS. In detail, our findings show a M_W_ of 3.7 and 5.0 MDa for AAV8 VLPs either lacking or carrying an engineered genome, respectively.

## 1 INTRODUCTION

Gene therapy (GT) aims to treat, or cure, a specific disease whose origin is linked to mutation(s) or incorrect expression of a gene.^1^ The approach involves delivering an engineered genomic load to add, replace, or interfere with the genetic layout of a cell in question to modify and correct it.^2^ The genomic cargo delivery relies on specific vehicles, which are generally grouped into viral and non‐viral vectors based on their origin. Both groups have advantages and limitations; non‐viral vectors are usually easier to synthesize and assemble than viral ones but have lower transduction efficiency.^3^ Instead, viral vectors can efficiently transport their cargo to the target but are often hindered by higher immunogenicity.^4^

In the vector‐mediated gene therapy realm, adeno‐associated virus (AAV) is the leading vehicle thanks to its high efficiency of transduction and low immunogenicity, as demonstrated by the growing number of clinical trials based on this delivery system.^5^, ^6^, ^7^, ^8^

AAV is a member of the family of *Parvoviridae*, genus *Dependoparvovirus*. It can accommodate up to 4.7 kb of single‐stranded DNA (ssDNA) in a non‐enveloped, proteinaceous capsid of approximately 26 nm in diameter. According to several sources, a molar ratio of 1:1:10 of the 60 viral proteins VP1, VP2, and VP3 arranged in a T = 1 icosahedral symmetry forms the protein shell.^4^, ^5^, ^9^, ^10^, ^11^ In nature, the AAV group is composed of 13 natural serotypes, each with a preferred tropism toward a specific tissue, thus making AAV a robust system for the transduction of specific cell types.^5^, ^12^

In this study, AAV serotype 8 (AAV8) has been used to produce virus‐like particles (VLPs). VLPs are proteinaceous “empty” shells derived from viruses, which can be used as vaccine^13^, ^14^ or as viral vector for the delivery of genetic material or other therapeutics,^15^, ^16^, ^17^ making them a highly adaptable platform.^18^ They are non‐infectious because the original viral genome is no longer present; instead, engineered genetic material can be encapsulated. In our study, two AAV8 preparations were available for analysis: (i) a so‐called “empty” one composed of solely the proteinaceous capsid lacking any genomic cargo and (ii) a so‐called “filled” preparation with an encapsulated engineered (non‐viral) genome. These two types of preparations were analyzed via native nano‐ES (electrospray) gas‐phase electrophoretic mobility molecular analysis (nES GEMMA) and with a native nano‐ESI (electrospray ionization) quadrupole reflectron time‐of‐flight mass spectrometry (nES QRTOF MS).

The nES GEMMA device, as first described by Kaufman et al.,^19^ is a suitable platform for analyzing proteins, viruses, VLPs, liposomes, and several nanoparticles and bionanoparticles, as demonstrated by various studies.^20^, ^21^, ^22^, ^23^, ^24^, ^25^ The system is also known under the name of differential mobility analyzer (nES DMA), macro ion mobility spectrometer (macroIMS), LiquidScan ES, or scanning mobility particle sizer (SMPS), all describing the same concept — the size‐separation of surface‐dry, single‐charged (bio‐)nanoparticles in the gas‐phase at atmospheric pressure.

The nES GEMMA device is composed of three distinct units: (i) The nES source electrosprays the analytes dissolved in a volatile electrolyte solution, while charge equilibration for the production of a polydisperse aerosol of single‐charged ions is achieved through a bipolar atmosphere generated by a radioactive source (e.g., ^210^Po α‐particle emitter),^26^ a soft X‐Ray charger,^27^, ^28^ or an alternating bipolar corona discharge process.^29^ (ii) A differential mobility analyzer unit, where a laminar sheath flow of particle‐free, dried air at atmospheric pressure, and an orthogonal tunable electric field, are used to achieve nanoparticle separation (i.e., gas‐phase electrophoresis). The generated monodisperse (monomobile) aerosol is introduced in (iii), a condensation particle counter, where its elements (i.e., the bionanoparticles) act as condensation nuclei for droplet formation due to the supersaturated atmosphere of either n‐butanol or water. By means of a laser beam, the formed μm‐sized droplets were detected as well as counted after size separation allowing particle‐number concentrations to be obtained. It is important to note that particle size determination occurs in the gas‐phase at atmospheric pressure. Hence, nES GEMMA yields the surface‐dry particle's size diameter (electrophoretic mobility diameter, EMD).^30^, ^31^ Therefore, for AAV8 VLPs, given the approximately spherical shape (i.e., icosahedral) and non‐enveloped origin (proteinaceous‐only capsid), the detected EMD can be directly correlated to the nanoparticles' diameter. Hence, the obtained EMD can be easily converted with good approximation in a molecular weight value thanks to an EMD/M_W_ correlation based on VLPs MS‐derived data.^22^, ^23^ This technology's significant advantages are manifested by its simple use, low operating cost, low sample usage, and well‐defined results, especially for analytes with a molecular weight (M_W_) ranging from kDa to several MDa. Particle size range coverage is defined mainly by the sheath gas flow rate in the DMA, spanning from 1.95 to 64.4 nm for the highest setting (i.e., 15.0 Lpm, liter per minute), or up to 181.1 nm with the lowest one (i.e., 2.5 Lpm) in the applied instrument.^22^

Native MS proved to be essential, and capable, for studying non‐covalent protein‐ligand^32^ and protein–protein interactions,^33^ protein complexes,^34^ and supramolecular protein structures like viruses^23^, ^35^ and VLPs.^22^, ^34^, ^35^, ^36^ The main challenge for this MS approach is to desorb/ionize and detect the multiple‐charged analytes while preserving their labile non‐covalent interactions and structure. Nonetheless, several VLPs have been successfully analyzed by employing commercially available MS instrumentation, such as nESI orbitrap^37^, ^38^, ^39^, ^40^ and nESI charge detection mass spectrometry (CDMS).^41^, ^42^ In our case, we employed a Synapt G1 (Waters Manchester, UK) modified by MS Vision (Almere, The Netherlands) to study AAV8 nanoparticles. The instrument is equipped with a nano‐electrospray ionization source for the production of multi‐charged bionanoparticles, and with several custom modifications to properly fine‐tune the necessary settings (e.g., application of collision and cooling gas, vacuum levels, and voltage settings) for successful analysis.^43^ Precise M_W_ determination can be assessed via deconvolution of the charge state assignment of the detected bionanoparticles.

In this manuscript, our focus is to combine native nES GEMMA and native nESI QRTOF MS data as well as an EMD/M_W_ correlation to expand, with great accuracy, the knowledge about the nanoparticles' size, sample quality, and molecular weight of AAV8 nanoparticles, either carrying or lacking an engineered genomic cargo.

## 2 MATERIALS AND METHODS

### 2.1 Chemicals, electrolyte solutions, and buffers

Ammonium acetate (NH_4_OAc, ≥99.99%) and ammonium hydroxide (ACS reagent) were both purchased from Sigma‐Aldrich (Steinheim, Germany). The nES GEMMA electrolyte solution was prepared by dissolving 40mM of ammonium acetate with water of ultra‐high quality (UHQ) delivered by a Simplicity UV apparatus (18.2 MΩ × cm at 25°C, Millipore, Billerica, MA, USA). The solution was adjusted to pH 7.0 with ammonium hydroxide and filtered through a surfactant‐free cellulose acetate membrane with 0.20‐μm pore size syringe filters (Sartorius, Göttingen, Germany).

### 2.2 Samples

HEK (human embryonic kidney) cell produced, purified AAV8 VLP samples were provided by Baxalta Innovations (Orth/Donau, Austria, part of Takeda). Two different batches were provided: (i) so‐called “empty” AAV8 VLPs (3776 μg/ml, i.e., 7.3 × 10^14^ capsids/ml) with 93% of capsids not carrying any genomic information and (ii) so‐called “filled” AAV8 VLPs (85 μg/ml, i.e., 1.6 × 10^13^ capsids/ml), where 66% of all capsids were carrying the full genomic load (an engineered genome). The percentage of capsid filling was assessed via transmission electron cryomicroscopy (CryoTEM).

For nES GEMMA as well as nESI QRTOF MS analysis, a buffer exchange step against 40‐mM NH_4_OAc was carried out employing 10‐kDa MWCO centrifugal filters (polyether sulfone membrane from VWR, Vienna, Austria). After three spin filtration repetitions (9.0 × 10^3^ g for 5 min each), the retentate was collected. Based on asymmetric flow field‐flow fractionation (AF4 also known as AFFFF) analysis, the estimated final sample concentration for “empty” AAV8 VLPs was 22 μg/ml, while for “filled” AAV8 VLPs it valued 8.5 μg/ml.

### 2.3 nES GEMMA

nES GEMMA analyses were carried out on a TSI Inc instrument (Shoreview, MN, USA), which consisted of a nano‐electrospray unit with a charge reduction source (model 3480 including a ^210^Po charge equilibration device), an electrostatic classifier equipped with a nano‐differential mass analyzer (nano‐DMA; model 3080) and an *n*‐butanol driven ultrafine condensation particle counter (CPC; model 3025A) for particle detection. For the spraying process, the nES unit is equipped with a 24 cm long, polyimide coated, fused‐silica capillary with an inner diameter of 25 μm (Polymicro Technologies, a subsidiary of Molex; Phoenix, AZ, USA). The capillary is manually cut and tapered with a home‐built grinding machine based on the work of Tycova et al.^44^

Nanoparticle separation and detection were achieved by using the following settings: The filtered airflow on the nES generator was set to 1.6 × 10^−5^ m^3^/s (1 Lpm), the CO_2_ gas flow to 1.6 × 10^−6^ m^3^/s (0.1 Lpm, 99.5% from Messer, Gumpoldskirchen, Austria) and the differential capillary pressure at 27.58 kPa (four pounds per square inch differential, PSID). Capillary conditioning was performed by pre‐spraying each sample for at least 3 min before starting any measurement. Capillary rinsing was performed by infusing the electrolyte solution until no signal from the previous sample was detectable. The sample was infused at a flow rate of approx. 70 nl/min. The capillary tip voltage was set to have a stable Taylor cone (approximately 2‐kV voltage resulting in approximately −380‐nA current). The electrostatic classifier was set in automatic scanning mode (up scan time for voltage adjustment 120 s, retrace time to initial voltage values 30 s) with a sheath gas flow rate of 2.5 × 10^−4^ m^3^/s (15 Lpm), which yielded a range of measurable electrophoretic mobility (EM) diameters between 2 and 65 nm. A total of 10 scans for each sample was used to generate a median spectrum. Mathematical and statistical calculations on the nES GEMMA spectra were made using OriginPro 9.1 (OriginLab, Northampton, MA, USA).

### 2.4 nES QRTOF MS

A Synapt G1 (Waters, Manchester, UK) was modified by MS Vision (Almere, The Netherlands) in order to maximize ion transmission for native nESI MS in the kilodalton to megadalton range. This was achieved by (i) increasing the operating pressure of the first vacuum stage (source region) by a manually controlled throttle valve (i.e., 5 to 10 mbar); (ii) fine tuning of the second vacuum stage (transfer pressure region) by fitting a sleeve that restricts pumping of the gas entering from the source region; (iii) installation of a 32 kDa quadrupole mass filter; (iv) amenities to bleed cooling gas like Ar of Xe into the ion mobility stage of the instrument at optimal pressures for cooling and desolvation as well as for independent control of trap and transfer collision cell pressures; (v) customized data acquisition settings (profile binning) and pusher pulse interval (i.e., 128 μs) were adjusted to improve ion detection at ultrahigh mass range. Sample introduction was performed by a nESI source employing manually opened in‐house pulled spray capillary. Sample concentration was chosen in order to achieve best results (i.e., avoid clogging of the tip and allow extremely long acquisition time). Spray capillary voltage was set to obtain ideal spraying condition (i.e., ranging between 1 to 2.5 kV). Gas pressures in the ion source region and in the ion mobility chamber (specifically the TriWave™ cell) before the orthogonal RTOF were finely tuned in order to increase ion transmission. Moreover, a relative high collision induced dissociation voltage (ranging up to 90 V) was applied to increase desolvation and optimize transmission efficiency.^45^, ^46^ The investigated mass range was between m/z 1000 and 40,000 in the positive ion mode. Mass spectra were analyzed using MassLynx (Waters, Manchester, UK) and OriginPro 9.1 (OriginLab, Northampton, MA, USA).

## 3 RESULTS AND DISCUSSION

Focusing on the molecular weight determination of (bio‐)nanoparticles in general and AAV serotype 8 in particular, we took interest in AAV8 VLPs either carrying or lacking engineered genomic material in their native state as enclosed proteinaceous capsid in the current manuscript. Instrumentation that was already fitted, or modified, for the purpose of studying protein complexes in their native conformation, such as nES GEMMA and nESI QRTOF MS was employed. The results obtained from nES GEMMA were correlated with a literature based EMD/M_W_ correlation for VLPs and used to aid the interpretation of native MS data.

### 3.1 Native nES GEMMA analysis of AAV8 VLPs

**FIGURE 1:**
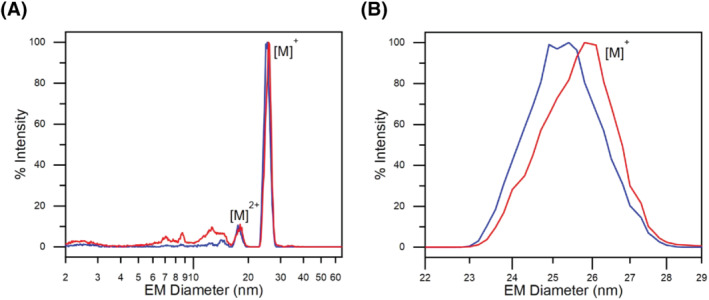
Native nano‐electrospray gas‐phase electrophoretic mobility molecular analyzer (nES GEMMA) analysis of “empty” (blue profile) and “filled” (red profile) AAV8 VLPs. (A) The entire analyzed range is presented. (B) Magnification of the electrophoretic mobility diameter (EMD) range from 22 to 29 nm of panel (A)

Gas‐phase electrophoresis of several VLPs—based on bacteriophages, a norovirus serotype, hepatitis B virus, cowpea mosaic virus and a human rhinovirus—yielding surface dry particle EMDs has already been described.^22^, ^23^, ^42^, ^47^ In addition, AAV8 VLPs have likewise been measured via gas‐phase electrophoresis as described in a previous work focusing on VLP aggregation (submitted manuscript). Focusing on the molecular weight of bionanoparticles in the current manuscript, Figure 1A depicts the nES GEMMA spectra of “empty” (blue profile) and “filled” (red profile) AAV8 VLPs in their native state. In order to better appreciate the fine difference between the two preparations, Figure 1B shows the magnification between 22‐ and 29‐nm EMD of Figure 1A. The slight difference in the EMD size is enough to discriminate between the two sample preparations. To confirm this observation, a statistical evaluation over more than 5000 capsids per preparation (*n* = 3 independent nES GEMMA measurements, each) was made. Results show an average EMD of 25.10 ± 0.18 nm and 25.93 ± 0.07 nm for “empty” and “filled” AAV8 VLPs, respectively. The difference in EMD is based on the stabilizing effect promoted by the genomic material inside the capsid of “filled” AAV8 VLPs. Lack of the genomic material as a scaffold in the working environment condition of nES GEMMA causes the partial shriveling of the capsid, hence reducing its EMD.

### 3.2 nES GEMMA‐based molecular weight determination

**FIGURE 2:**
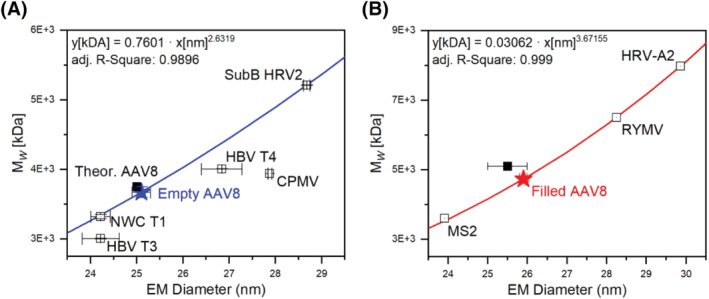
Electrophoretic mobility diameter (EMD)/M_W_ correlations for (A) “empty” virus‐like particles (VLPs) and (B) “filled” VLPs (i.e., intact virus). Readapted with permission from Weiss et al.^22^, ^23^ Legend: NWC T1, Norovirus West Chester T1 VLP; HBV T3, hepatitis B virus T3 VLP; HBV T4, hepatitis B virus T4 VLP; CPMV, cowpea mosaic virus VLP; SubB HRV2, subviral B particle of human rhinovirus 2; MS2, bacteriophage MS2; RYMV, rice yellow mottle virus; HRV‐A2, human rhinovirus serotype 2

The correlation between EMD data, obtained from nES GEMMA measurements, and the M_W_ of several VLPs or virus particles, either from literature or measured via MS instrumentation, has already been reported.^22^, ^23^ The application of the EMD/M_W_ correlations provided in the studies mentioned above is presented in Figure 2. The data produced via nES GEMMA analysis for AAV8 generate M_W_ of 3670 ± 69 kDa (Figure 2A) and 4751 ± 47 kDa (Figure 2B) for “empty” and “filled” capsids, respectively. A summary of M_W_ values is presented in Table 1.

**TABLE 1:** Measured size data, theoretical M_W_ data, derived M_W_ data and mass spectrometric data of empty and filled AAV8 VLP preparation

	AAV8 VLP preparations	
Investigative approach:	Empty	Filled
nES GEMMA EMD data (nm)	25.10 ± 0.18	25.93 ± 0.07
Theoretical M_W_ based on 1:1:10 VPs ratio (kDa)^a^	3746	5076
Theoretical M_W_ based on SDS‐PAGE (kDa)	3658	4988
EMD/M_W_ correlations (kDa)	3670 ± 69	4751 ± 47
Native MS (kDa) (for *n* = 161 charges)	3710	5005

Abbreviations: EMD, electrophoretic mobility diameter; nES GEMMA, native nano‐electrospray gas‐phase electrophoretic mobility molecular analyzer; MS, mass spectrometry.

^a^ Based on the following M_W_: VP1 81 kDa; VP2 65 kDa; VP3 60 kDa.^48^

The M_W_ resulting from the EMD/M_W_ correlation for the “empty” capsid highly correlates when compared with data based on crystal structural studies^48^ (i.e., 3746 kDa, difference 2.1%) or based on gel electrophoretic data (i.e., 3658 kDa, difference 0.3%). In both cases, the molecular weight of VP1, VP2, and VP3 is multiplied by the capsid protein ratio; in the first case, with data available in the literature,^48^ while in the second case, the protein ratio is estimated on the basis of SDS‐PAGE experiments (data not shown). For the “filled” capsid, the resulting M_W_ (i.e., 4751 ± 47 kDa) is fitting to a lower degree to the expected value. Precisely, by adding the M_W_ of the encapsulated genome (i.e., 1330 kDa) to the M_W_ of the “empty” capsid (3658 kDa), a total molecular weight of 4988 kDa is calculated. This results in a mass difference of 4.75% to the experimental value of 4751 ± 47 kDa as obtained via native nES GEMMA measurements and the application of the corresponding correlation.

### 3.3 Native nES QRTOF MS analysis of AAV8 VLPs

The analysis of VLPs in their native state is a delicate and laborious job. In this study, megadalton‐range species were targeted, which further increased the analytical challenges. The biggest challenge for analyzing such massive species is the passage of desorption/ionization region and transfer into the vacuum part of the mass spectrometer. Parameters like sample concentration, quality and shape of the capillary tip, and the mass spectrometer's pressure in the first two differentially pumped vacuum stages greatly influenced the outcome. The response to each of these settings was rather drastic, to the magnitude where analytes' detection was either successful or not.

**FIGURE 3:**
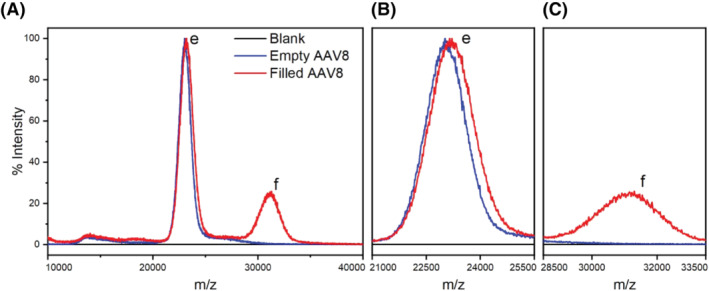
Native positive ion mass spectra of “empty” (blue profile) and “filled” (red profile) AAV8 VLPs. The “empty” VLPs preparation shows a dominant peak *e* assigned to monomeric “empty” capsids. The “filled” VLPs preparation contains the shared peak *e* and a second peak *f* assigned to “filled” capsids. The figure comprises (A) the entire m/z range and the magnification of the range for (B) peak *e* and (C) peak *f*

In Figure 3A, the positive ion mass spectra of AAV8 VLPs, either “empty” (blue profile) or “filled” (red profile), are shown. The blue profile shows a single dominant peak with an apex center at 23,047 m/z. At the same time, the red profile shows two peaks, a dominant one at 23,205 m/z and a second at 31,092 m/z. Although charge resolution was not achieved and hence no molecular weight determination based on peak charge assignment was possible, it is highly plausible that the detected peaks belong to “empty” (label *e*) and “filled” (label *f*) AAV8 VLPs. Further support comes from the presence of a shared peak between the two preparations (i.e., label *e*, Figure 3B) because the “filled” AA8 VLPs preparation contains at least 33% of AAV8 VLPs lacking genomic cargo.

Consequently, the peak labeled *f*, detected only in the “filled” AAV8 preparation (Figure 3C) represents the portion of capsids carrying the genomic cargo. Moreover, although the concentration of “filled” capsids in the sample exceeds “empty” ones' concentration, this is not reflected in the mass spectra. This discrepancy can be explained by a lower transmission efficiency due to the increased mass of the analytes for “filled” VLPs, and/or by an uneven desorption/ionization response between the two VLP species. Lastly, as already mentioned, the lack of charge resolution does not allow to calculate the precise molecular weight of either capsids' preparations. Besides the high amount of resolving power required to obtain charge distribution accurately, it is highly plausible that capsid heterogeneity plays a role in this matter, as already investigated by Snijder et al.^39^ To overcome this issue, a method that relies on the results generated by nES GEMMA and literature‐based EMD/M_W_ correlations is proposed to estimate the charges enveloping the capsids.

### 3.4 Combining native nES GEMMA and native nESI QRTOF MS data for M_W_ determination of AAV8 VLPs

For the native MS analysis, as already pointed out, charge resolution was not achieved. Therefore, to estimate the M_W_ of the detected analytes, the following method is proposed: Because the encapsulated genome's size is known and based on the capsids' weight obtained from the EMD/M_W_ correlations mentioned earlier, an accurate estimation of the number of charges enveloping the capsids can be made. Therefore, based on (i) the assumption that the apex center of peak *e* (i.e., 23,047 m/z) in Figure 3A,B is generated only by “empty” monomeric VLP species, and (ii) given the M_W_ of 3670 ± 69 kDa obtained from nES GEMMA data as described before is valid, only ions with a number of positive charges ranging from 157 to 162 would generate analytes whose M_W_ could fit the EMD/M_W_ correlation. Consequently, the peak *e* yields a M_W_ of 3676 ± 58 kDa as an average of calculated values for all charge numbers between 157 and 162 positive charges.

Because the genome encapsulated in the proteinaceous capsid is shielded from the external environment, we suppose that it does not affect the number of charges enveloping the capsid but only its molecular weight. To support this claim, the same range of positive charges assigned to peak *e*, have been applied to peak *f* (i.e. 31,092 m/z, Figure 3C). Thus, a M_W_ of 4959 ± 78 kDa is obtained. As a result, this calculation highly correlates with the molecular weight for ‘filled’ VLPs obtained from the EMD/M_W_ correlation (i.e., 4751 ± 47 kDa, difference 4.4%) or from the expected theoretical M_W_ mentioned before (i.e., 4988 kDa, difference 0.6%). Moreover, because the molecular weight of the encapsulated genome is known, its size can be used to narrow down the range of possible charges of the capsid by comparing the difference in weight between “filled” and “empty” VLPs. As a result, a total of 161 positive charges, for both “empty” and “filled” VLPs, is the value that produces the lowest difference to the genome's molecular weight (i.e., 0.4%).

## 4 CONCLUDING REMARKS

In this study, nES GEMMA and native MS spectrometry were applied to analyze and characterize AAV8 VLPs either lacking or carrying a non‐viral engineered genomic cargo. The nES GEMMA instrumentation can determine the dry‐surface diameter of particles in the nanometer range. This makes nES GEMMA an ideal device for the characterization of nanoparticles and bionanoparticles, for instance, VLPs. Native MS aims to preserve non‐covalent interaction, allowing the characterization of proteinaceous complexes such as viral capsids like the one presented in this study. Both techniques do present some limitations; nES GEMMA cannot directly determine the exact molecular weight of the detected AAV8 nanoparticles but only infer it from EMD/M_W_ correlations based on other data (e.g., SDS‐PAGE or MS of the individual capsid proteins). Native MS instead generates mass‐over‐charge results but suffers from low ion transmission efficiency at very high molecular weight and deconvolution challenges.

The EMD obtained from native nES GEMMA analysis results in 25.10 ± 0.18 nm and 25.93 ± 0.07 nm for “empty” and “filled” AAV8 VLPs, respectively. Based on EMD/M_W_ correlations, these results directly translate to the molecular weights of 3670 ± 69 kDa and 4,751 ± 47 kDa for “empty” and “filled” capsids, respectively.

Although native nESI QRTOF MS was successful for detecting both types of capsid preparations, but charge resolution for exact molecular weight determination was not achieved. To overcome this issue, the data inferred by the EMD/M_W_ correlations and the size of the encapsulated genome were used to estimate with high accuracy the number of charges enveloping the capsids, thus deriving the molecular weight of both “empty” and “filled” VLPs. The genomic cargo, since encapsulated in, and protected by, the proteinaceous capsid, is expected to influence solely the overall molecular weight and not the number of charges surrounding the capsid. Therefore, based on the proposed methodology, the detected peaks' apexes are estimated to carry 161 charges, resulting in a M_W_ of 3710 kDa (1.1% difference from the EMD/M_W_ correlation based value) and 5005 kDa (5.4% difference from the EMD/M_W_ correlation based value) for “empty” and “filled” VLPs, respectively. These findings corroborate the expected values derived from theoretical calculation and nES GEMMA EMD/M_W_ correlations, thus further consolidating the fidelity of EMD/M_W_ correlations. Based on our findings, we were able to demonstrate that the combination of native nES GEMMA and native nESI QRTOF MS is very powerful, enabling the in‐depth interpretation of data derived from each of these two analysis methods alone to a much higher level of detail (refer to Table 1 for an overview on obtained values).

Noteworthy, SDS‐PAGE analysis aimed to determine the viral protein's ratio provided by the manufacturing company of the AAV8 VLPs (data not shown) as well as from the work of Snijder et al.,^39^ indicate that the VP's ratio is different from the ratio largely listed in the literature, thus indicating that AAV8 VLP vectors are not strictly constrained to the 1:1:10 VPs ratio. This protein ratio heterogeneity might influence, for example, host cell infection and affecting accurate charge detection for molecular weight determination. It is undeniable that further studies to unveil more details about these viral vectors are required, especially as instrumentation (e.g., high‐resolution DMAs) and methods for native MS (e.g., CDMS) at high Mw species advance.

## Data Availability

No additional data are available.
